# Niche segregation in two closely related species of stickleback along a physiological axis: explaining multidecadal changes in fish distribution from iron-induced respiratory impairment

**DOI:** 10.1007/s10452-012-9395-y

**Published:** 2012-04-21

**Authors:** Wilco C. E. P. Verberk, Piet J. J. van den Munckhof, Bart J. A. Pollux

**Affiliations:** 1 Department of Animal Ecology and Ecophysiology, Institute for Water and Wetland Research, Radboud University, Toernooiveld 1, 6525 ED Nijmegen, The Netherlands; 2 Bargerveen Foundation, P.O. Box 9010, 6500 GL Nijmegen, The Netherlands; 3 Staatsbosbeheer, Regio Zuid, Spoorlaan 444, 5038 CH Tilburg, The Netherlands; 4 Experimental Zoology Group, Department of Animal Sciences, Wageningen University, De Elst 1, 6708 WD Wageningen, The Netherlands; 5 Present Address: Marine Biology and Ecology Research Centre, University of Plymouth, Davy Building, Drake Circus, Plymouth, PL4 8AA UK

**Keywords:** Distribution, Heavy metals, Toxicity, Life-history strategy, Oxygen, Physiological tolerance

## Abstract

Acute exposure to iron can be lethal to fish, but long-term sublethal impacts of iron require further study. Here we investigated whether the spatial and temporal distribution (1967–2004) of two closely related species of stickleback matched the spatial distribution of iron concentrations in the groundwater. We used the ‘Northern Peel region’, a historically iron-rich peat landscape in The Netherlands as a case study. This allowed us to test the hypothesis that niche segregation in two closely related species of stickleback occurred along a physiological axis. Patterns in stickleback occurrence were strongly associated with spatial patterns in iron concentrations before 1979: iron-rich grid cells were avoided by three-spined stickleback (*Gasterosteus aculeatus*, Linnaeus 1758) and preferred by nine-spined stickleback (*Pungitius pungitius*, [Linnaeus, 1758]). After 1979, the separation between both sticklebacks became weaker, corresponding to a decreased influence of local groundwater on stream water quality. The way both species changed their distribution in the field provides a strong indication that they differ in their susceptibility to iron-rich conditions. These observed differences correspond with differences in their respiration physiology, tolerance of poor oxygen conditions and overall life-history strategy documented in the literature. Our results exemplify how species can partition niche along a non-structural niche axis, such as sublethal iron-rich conditions. Other fish species may similarly segregate along concentration gradients in iron, while sublethal concentrations of other metals such as copper may similarly impact fish via respiratory impairment and reduced aerobic scope.

**Electronic supplementary material:**

The online version of this article (doi:10.1007/s10452-012-9395-y) contains supplementary material, which is available to authorized users.

## Introduction

Metal toxicity can cause a decline or loss of fishery resources (Carpenter 1927; Spry and Wiener 1991). Human activities may cause local metal contamination (e.g. waste effluent associated with mining activities; Gundersen et al. 2001), but metal contaminations can also be geographically widespread in nature. For example, acidification effects may enhance the bioaccumulation of metals (Wiener 1987). For the metal iron, high concentrations are a common, naturally occurring phenomenon. Groundwater in contact with iron-rich deposits, such as glauconite, will be enriched, resulting in iron-rich seepage at depressions in the landscape such as river meanders (Lucassen et al. 2006) or near geological faults (van den Munckhof 2000).

**Fig. 1:**
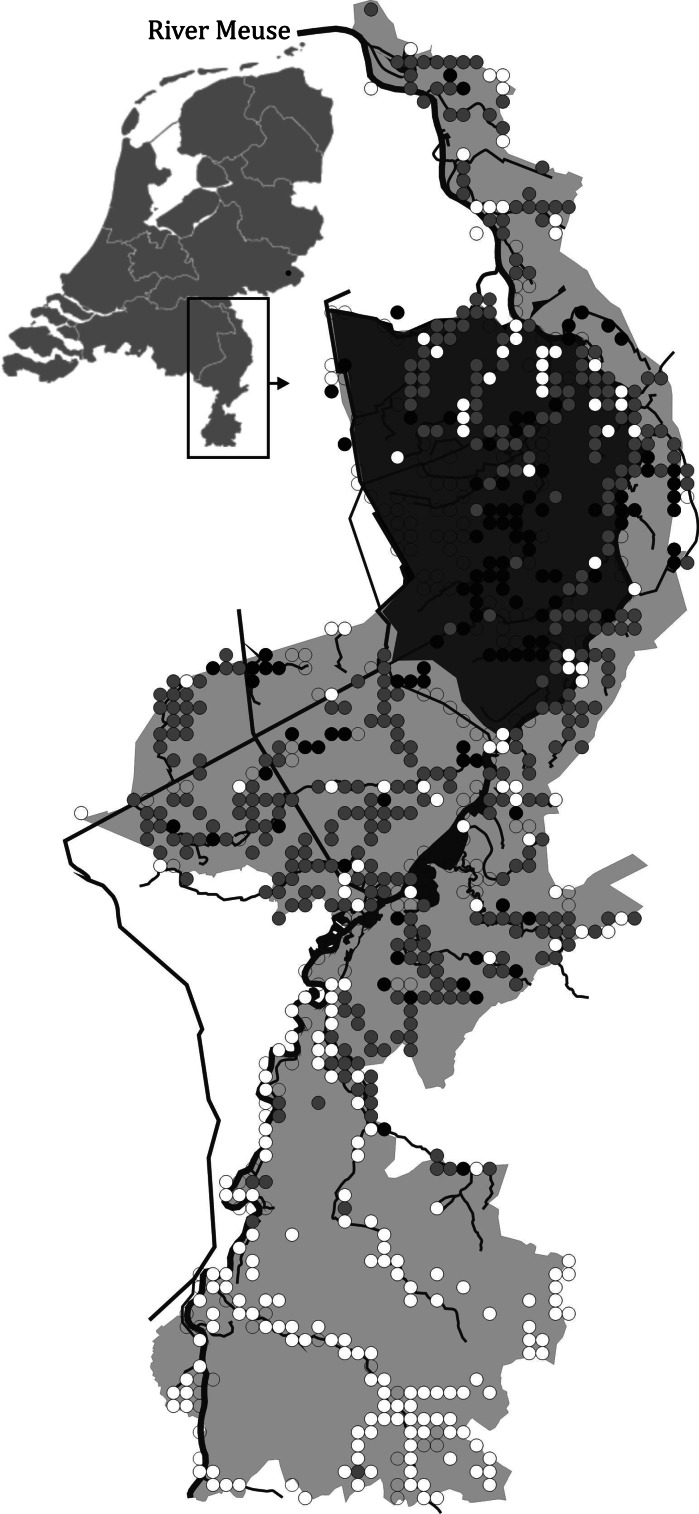
Distribution of the three-spined stickleback (*Gasterosteus aculeatus*; *white*) and nine-spined stickleback (*Pungitius pungitius*; *black*) in the province of Limburg, The Netherlands. Co-occurrences of both sticklebacks are indicated in *light grey*, while *open circles* indicate an absence of both species. Chi-square statistics indicate that both species co-occur more often than expected by chance (χ_1,981_^2^ = 6.80; *P* = 0.0091). *Inset* of The Netherlands shows the province of Limburg in which the study region (the Northern Peel region) is shown in *dark grey*

Iron but also copper and zinc are believed to act primarily on the gills, impairing respiration and causing death through asphyxiation (Jones 1939; Dalzell and Macfarlane 1999; Waser et al. 2010). Iron has been shown to precipitate on the gills, physically impairing oxygen uptake; the absorption and subsequent bioaccumulation of iron in a fish’s organs is less likely to be an important toxic pathway (Dalzell and Macfarlane 1999). Acute exposure to iron was found to be lethal in brown trout, *Salmo trutta*, but long-term sublethal impacts of iron require further study (Dalzell and Macfarlane 1999). Iron can be beneficial for freshwater wetlands as free Fe can bind phosphate and hence inhibit eutrophication (Smolders et al. 2001; Lucassen et al. 2006), which makes it important to study potential toxic effects on wildlife. Here, we investigated the spatial and temporal distribution of two closely related species of stickleback to see whether distribution patterns match the known distribution of iron-enriched streams. We use an extensive database containing distribution records for both species in streams in the Dutch province of Limburg where both species occur in sympatry (Fig. 1) and which uniquely covers a long period (1967–2004). We focus on the ‘Northern Peel region’, a historically iron-rich peat landscape in the Province of Limburg, The Netherlands (van den Munckhof 2000), as a case study to investigate potential long-term effects of iron on fish.

Both stickleback species have been found to co-occur more often than expected by chance in streams (Copp and Kováč 2003; Fig. 1). Habitat structure, including spatial differences in flow and substratum, is generally held to be important for the coexistence of stream fishes (Gorman and Karr 1978). Although the nine-spined stickleback does exhibit a slight preference for aquatic plants and dense mats of filamentous algae in muddy areas (Lewis et al. 1972; Hart 2003), little evidence exists for microhabitat competition between both species (Copp 1992; Copp et al. 1998). The fundamental problem is that one can always maintain that the relevant, that is, segregating niche axis has not been measured, given the multidimensional nature of the niche concept (Hutchinson 1959). In the case of sticklebacks, differences between species may be on a physiological axis. Jones (1939) concluded that the acute toxicity of ferric chloride solutions (FeCl_3_) on the three-spined stickleback (*Gasterosteus aculeatus*, Linnaeus 1758) is due to their acidity and that the ferric ion (Fe^3+^) has little or no lethal effect. Although not fatal, iron-induced respiratory impairment may decrease aerobic scope (the capacity to perform aerobic activity), which likely impacts growth, reproduction and population dynamics in the long term (Pauly 2010). In this respect, differences in rates of oxygen consumption between both species are highly relevant. Although similar toxicological studies on the closely related nine-spined stickleback (*Pungitius pungitius*, [Linnaeus, 1758]) are currently not available, ecological studies unequivocally show that the nine-spined stickleback has a superior tolerance to poor oxygen conditions (Bănărescu and Paepke 2002) and has a far lower oxygen consumption (Lewis et al. 1972). Since iron toxicity acts through respiratory impairment, it follows that nine-spined stickleback should be more tolerant to higher iron concentrations than three-spined stickleback. This idea is indirectly corroborated by a recent study by Waser et al. (2010), who showed that lower rates of oxygen consumption across different populations of nine-spined stickleback corresponded to higher survival rates when exposed to high copper concentrations.

The aim of the present study was to examine potential long-term effects of iron on the distribution patterns of fishes that differ in physiological tolerance. We assess this in two ways: first, we examine whether the spatial distribution of three- and nine-spined stickleback corresponds to iron-poor and iron-rich habitats, respectively. Based on higher tolerance levels to poor oxygen conditions of nine-spined stickleback, we predict that iron-rich sites will be primarily occupied by nine-spined stickleback. Secondly, we examine whether temporal shifts in the spatial distribution of the two study species correspond to changes in iron levels in the surface waters. Around 1979, the influence of iron-rich groundwater on surface waters in our study area decreased significantly after many land consolidation and stream normalisation projects were completed (Soesbergen et al. 1990; Verberk et al. 2004a). We predict that species’ distribution patterns shifted during this period, marked by a range expansion of three-spined stickleback corresponding with a decline in iron-rich surface waters. This study system thus presents us with a unique opportunity to study the effect of both *spatial* and *temporal* variation in iron concentration on the spatial distribution and range shifts of three- and nine-spined stickleback.

## Materials and methods

### Study area

The ‘Northern Peel region’ in the Provence of Limburg, The Netherlands (Fig. 1), is a sloping landscape with sandy deposits. Bogs have developed during the early Holocene, which once expanded across most of the region. At the beginning of the twentieth century, only a fraction of these bogs remained due to drainage, peat cutting and land cultivation, which already set in before the early middle ages. The area is traversed by several geological faults. Extensive glauconite deposits result in iron-rich seepage of shallow groundwater at these faults, leading to locally high iron concentrations in the surface waters (van den Munckhof 2000).

Around 1979, many land consolidation projects and stream normalisations were completed. These changes in local stream morphology increased the discharge capacity of these streams. To prevent streams from temporarily running dry, water from the River Meuse (poor in iron) was redirected and used to feed these streams to ensure continuous water supply for agricultural reasons (e.g. see Figure 1 in Pollux et al. 2006). This decreased the influence of local (iron-rich) groundwater on stream water quality and effectively homogenised existing gradients in stream water quality (Soesbergen et al. 1990; Verberk et al. 2004a).

Grid cells of 1 × 1 km were assigned to an ‘iron-poor’ and an ‘iron-rich’ group using a spatially interpolated map that shows where the iron content of shallow groundwater exceeds 10 mg per litre (iron-rich subregion) (see Figure S1). Grid cells located on the boundary of the iron-rich subregion were assigned to the same group as the upstream grid cell. This map was reported in van den Munckhof (2000) and based on the results of extensive geohydrological surveys (Homan 1974; Lekahena and Nelisse 1974; Nelisse 1974; Lekahena 1978). Grid cells follow the Dutch national ‘Rijksdriehoeks’ reference frame (de Bruijne et al. 2005).

### Fish records

Fish records were obtained from several sources with each record documenting the presence of a fish species in 1 × 1 km^2^ grid cells in a certain year. Historical literature (Cuppen 1977) and personal observations by the second author covered the complete time period (1967–2004), and these sources yielded 32 and 969 records of three-spined stickleback and nine-spined stickleback, respectively. Additional ‘stickleback’ records were obtained from the database of the ‘Natuurhistorisch Genootschap Limburg’, a Dutch nature organisation financed by the Province of Limburg aimed at promoting biological and geological research. This database is unique in its scale and sample coverage, containing over 80,000 records on fish occurrences from an area of approximately 2,200 km^2^ (Fig. 1). This database contained 2,197 ‘stickleback’ records, most of which (99.8 %) stemmed from the time period between 1990 and 2002. Of these 2,197 records, 474 originated from the Northern Peel region. Additional field sampling was undertaken between 2003 and 2004 to ascertain distribution limits of both species in the Northern Peel region, adding another 34 stickleback records. Table S1 gives an overview of the number of records for each of the species in each period.

Sampling effort is not documented as such in the database. For instance, samples that did not yield any species are not recorded in the database. Hence, we could not distinguish between those grid cells that were not sampled and those that were sampled but did not yield any stickleback. Although sampling effort was not directly available in the data set, multiple records for a single species indicate repeated sampling in that grid cell. On this basis, the minimum number of sampling occasions as a measure of sampling effort was derived for each grid cell for both time periods.

For the Northern Peel region, sampling effort was greater in the second period, when estimated both from the total number of sampling occasions (507 in the first period versus 1,117 in the second period) and from the total number of sampled grid cells (60 in the first period versus 240 in the second period). From these numbers, it follows that sampling intensity (number of sampling occasions averaged per grid cell) roughly halved (see Figure S2).

While this does not affect the spatial comparison (iron-rich versus iron-poor), it does put restrictions on the temporal comparison (period 1 vs. period 2), as an expanded distribution could in principle result from the greater spatial coverage of sampling. However, we can document temporal shifts in the distribution of the two species relative to each other and investigate how spatial patterns of co-occurrence have changed over time, because both species are easy to catch and identify and any sampling bias towards any one species is unlikely. Finally, calculating changes in the number of records can be done by applying a straightforward correction for sampling intensity (see Figure S4).

### Data analysis

Our analysis focused on the Northern Peel region and analysed stickleback distribution patterns using all stickleback records before 1979 (560 records) and after 1979 (949 records). Grid cells were categorised into three groups, based on whether only three-spined stickleback was recorded (group 1), only nine-spined stickleback was recorded (group 2), or both species co-occurred (group 3). Since the focus of this study was on changes in co-occurrence of the two species, grid cells that were not sampled and those that were sampled but did not yield any sticklebacks (‘empty’ grid cells) were excluded from the analysis. Given that sampling effort was not documented in the database (see above), a change in the number of these ‘empty’ grid cells could be also be caused by increases in non-focal species leading to spurious results if we had included them (e.g. gudgeon (*Gobio gobio* [L 1758]) increased strongly in the second period; Verberk et al. 2004b).

To test for differences in species (co-)occurrence across space and time, we performed a log-linear analysis on counts of grid cells in each of the three groups, with period (before/after 1979) and region (iron rich/iron poor) as explanatory variables. The log-linear analyses employed here are a specialised case of generalised linear models for Poisson-distributed data. This version of chi-square analysis, suited for 3-way contingency tables, is best suited to analyse the type of data reported here, which deals with counts and discrete blocks (Fienberg 1970). For each species, a similar analysis was performed using counts of fish records rather than counts of grid cells. The underlying idea is that multiple recordings of a species at a given grid cell may reflect higher abundance, more stable populations, or both, whereas this information is not reflected in counts of grid cells. For these analyses, fish records were grouped in two groups: those originating from grid cells exclusively occupied by one species (exclusive grid cells) and those originating from grid cells where both species co-occurred (shared grid cells).

## Results

### Grid cells

**Fig. 2:**
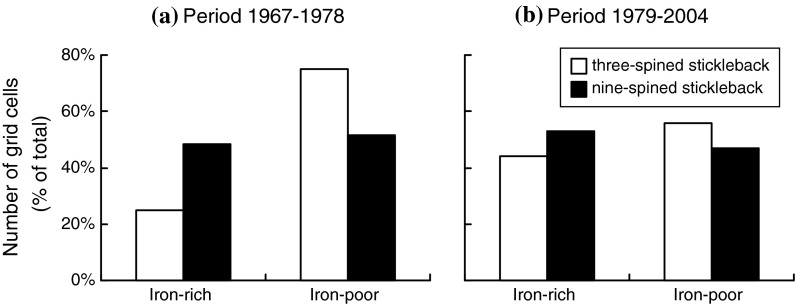
Number of grid cells as a percentage of the total number in the Northern Peel region in each of the two regions (iron rich and iron poor). The first (**a**) and the second periods (**b**) are shown separately. *Colours* depict three-spined stickleback (*Gasterosteus aculeatus*; *white*) and nine-spined stickleback (*Pungitius pungitius*; *black*)

Our study revealed marked differences in the distribution patterns of three- and nine-spined stickleback. In the first period (1967–1978), the nine-spined stickleback was the most wide ranging species occupying 58 grid cells, compared to only 34 grid cells for the three-spined stickleback (Table S1). The distribution of the three-spined stickleback was balanced towards the iron-poor region, while the nine-spined stickleback was equally distributed across both regions (Fig. 2a). In the second period (1979–2004), the number of occupied grid cells was almost equal for both species (118 cells for the three-spined stickleback and 145 cells for the nine-spined stickleback; Table S1) and the distribution of the three-spined stickleback was no longer balanced towards the iron-poor region (Fig. 2b).

The spatial pattern of co-occurrence of both species differed between regions, and this pattern changed over time (Group × Region × Period: *G* ^2^ = 32.98; *df* = 7; *P* < 0.0001; Table S1; Fig. S1). In the first period, the majority (~76 %) of iron-rich grid cells were occupied exclusively by nine-spined stickleback, while the majority of iron-poor grid cells were shared by both species. Virtually none of the grid cells (~3 %) were exclusively occupied by three-spined stickleback. In the second period, more grid cells (~17 %) were exclusively occupied by three-spine stickleback, and the difference between iron-rich and iron-poor grid cells was much reduced. The fraction of iron-rich grid cells occupied exclusively by nine-spined stickleback was markedly lower (reduced from ~76 to ~42 %).

### Records

**Fig. 3:**
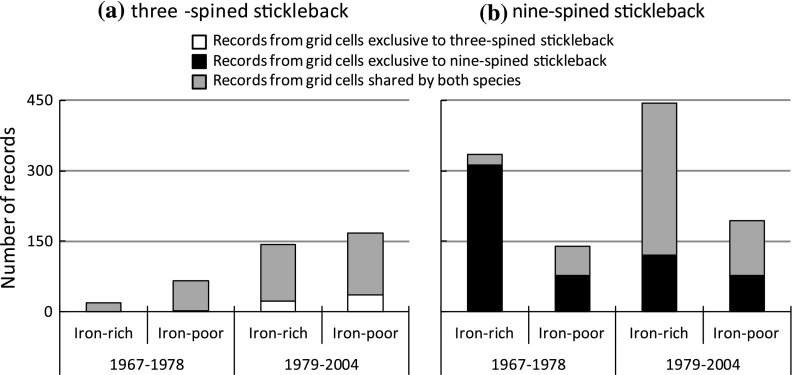
Number of records for (**a**) three-spined stickleback (*Gasterosteus aculeatus*) and (**b**) nine-spined stickleback (*Pungitius pungitius*), separated by region (iron rich and iron poor) and period (1967–1978 and 1979–2004). *Numbers* are further subdivided by *colours*, indicating whether records originate from grid cells that are shared by both species or from grid cells that are exclusive to one of the two species. *Colour* coding follows that of Fig. 1

The number of records from exclusive or shared grid cells was different between regions and changed over time for both the three-spined stickleback (Fig. 3a; Group × Region × Period: *G* ^2^ = 34.90; *df* = 4; *P* < 0.0001) and the nine-spined stickleback (Fig. 3b; Group × Region × Period: *G* ^2^ = 401; *df* = 4; *P* < 0.0001). The patterns in record counts were largely congruent with those for grid cells described above. The number of records for three-spined stickleback was much higher in the iron-poor region in the first period compared to the iron-rich region. Given that an almost equal number of grid cells was sampled in both regions, this indicates that three-spined stickleback avoided the iron-rich region (Fig. S4). This avoidance disappeared in the second period (Fig. 3a). The number of records for nine-spined stickleback was much higher in the iron-rich region in both periods, indicating a clear preference for iron-rich conditions in both periods (Fig. S4). However, in the second period, most records originated from shared grid cells as three-spined stickleback expanded its distribution into grid cells formerly exclusively occupied by nine-spined stickleback (Fig. S1; Fig. 3b).

## Discussion

Prior to 1979, patterns in stickleback occurrence were strongly associated with spatial patterns in iron concentrations. Iron-rich grid cells were avoided by three-spined stickleback (Figs. 2a, 3a) and preferred by nine-spined stickleback (Fig. 3b). After 1979, the separation between both sticklebacks became weaker. This weakened separation corresponded with a diminished influence of iron-rich seepage on local surface water quality, due to intake of (iron-poor) water from the River Meuse, allowing three-spined stickleback to spread out into the formerly iron-rich grid cells (Figs. 2b, 3a). The reported shifts in co-occurrence patterns are likely an accurate reflection of the true changes in species’ co-occurrence, given the commonness of both species and the high spatial coverage of the records (good spatial replication). The fact that both species are easy to catch and identify further increases the reliability of the distribution patterns and makes any sampling bias towards any one species unlikely.

Our study suggests that long-term exposure to iron need not be lethal (both species were found repeatedly in iron-rich grid cells). Nevertheless, the sublethal effects can affect fish distributions on the long term, possibly by reducing aerobic scope and altering competitive strength. Niche partitioning is considered an important mechanism to reduce competition between species and promote their coexistence (Gause 1934; Hutchinson 1959; MacArthur and Levins 1967). The differences in oxygen consumption suggest that the two species may differ along a physiological axis, where harsh habitats in terms of low oxygen or high iron concentrations may provide a refuge for the more tolerant species (see also Chapman et al. 2002; Yamanaka et al. 2007). This difference between both species in their physiological tolerance can explain the observed changes in co-occurrence after intake of water from the River Meuse. It also fits with other differences in their morphology, life-history and behaviour (Verberk et al. 2004a), reflecting the view that biological differences can be taken as a life-history strategy or a set of co-evolved traits that enable a species to deal with a range of ecological problems (Stearns 1976; Verberk et al. 2008).

The preference of nine-spined stickleback to reside and nest in dense vegetation, away from the bottom substratum where oxygen deficiency is likely more pronounced (Lewis et al. 1972; Hart 2003), may be a behavioural adaptation that complements its larger physiological tolerance. The three-spined stickleback, by contrast, is more likely to avoid, rather than cope with, sudden harsh conditions by swimming downstream (Maitland and Campbell 1992). Furthermore, the presence of the larger spines on the three-spined stickleback provides a more effective defence against (fish) predators, thereby lowering its dependence on vegetation cover while increasing its propensity to disperse in order to avoid (rather than cope with) the local harsh environmental conditions (Hoogland et al. 1956). Similarly, the high physiological tolerance of the nine-spined stickleback may come at a cost, as it has a lower overall reproductive allocation than the three-spined stickleback (Copp et al. 2002). This may place the nine-spined stickleback at a competitive disadvantage against the three-spined stickleback under benign conditions. Consequently, subtle differences in morphology and life-history strategy between three- and nine-spined sticklebacks may further help to explain the observed differences in occurrence in iron-rich and iron-poor regions. In the study region, nine-spined stickleback was historically more confined to marshy backwaters and smaller order streams (Verberk et al. 2004a, b). This difference in habitat choice between both species may very well reflect how niches are partitioned in a more intact stream valley. Simplification of stream valleys and loss of habitat heterogeneity have likely forced both species together.

In conclusion, our results exemplify how species can partition niche along a non-structural niche axis, such as sublethal iron-rich conditions. Interspecific differences in their susceptibility to iron-rich conditions correspond with documented differences between both stickleback species in their respiration physiology and tolerance of poor oxygen conditions. These interspecific differences match current and past stickleback distribution patterns and links observed temporal shifts in their distributions to changes in water quality. This study has wider relevance as other fish species may similarly segregate along concentration gradients in iron, while sublethal concentrations of other metals such as copper may similarly impact fish via respiratory impairment and reduced aerobic scope.

## Electronic supplementary material

Below is the link to the electronic supplementary material.

Supplementary material 1 (DOC 1,578 kb)
