# Supplementary material for: Niche segregation in two closely related species of stickleback along a physiological axis: explaining multidecadal changes in fish distribution from iron-induced respiratory impairment
Source: Aquat Ecol. 2012 Apr 21;46(2):241–8. doi: 10.1007/s10452-012-9395-y (PMC4431660; doi:10.1007/s10452-012-9395-y)
Supplement: Supplementary file 1 — Supplementary material 1 (DOC 1,578 kb) [file 10452_2012_9395_MOESM1_ESM.doc]

**Supplementary material**

Table S1

Overview of the number of grid cells occupied in the Northern Peel region by both species of sticklebacks in two periods (1967-1978; 1979-2003) and the number of records for each species. The area examined is situated in the northern part of the province Limburg, west of the river Meuse. The number of grid cells and records are reported for the region as a whole and for the part influenced by iron-rich groundwater (see Fig. 2.) separately. Grid cell and the records obtained in them fall into one of three categories; those with only threespine stickleback, those with only ninespine stickleback and those with both species (n.a.: not applicable).

|  |  | 1967-1978 |  |  |  | 1979-2003 |  |
| --- | --- | --- | --- | --- | --- | --- | --- |
|  | only threespine stickleback | only ninespine stickleback | threespine and ninespine stickleback |  | only threespine stickleback | only ninespine stickleback | threespine and ninespine stickleback |
| number of grid cells (km^2^) |  |  |  |  |  |  |  |
| Iron-rich region | 1 | 22 | 6 |  | 12 | 37 | 40 |
| Iron-poor region | 1 | 10 | 20 |  | 18 | 20 | 48 |
| Total region | 2 | 32 | 26 |  | 30 | 57 | 88 |
|  |  |  |  |  |  |  |  |
| number of records (threespine) |  |  |  |  |  |  |  |
| Iron-rich region | 1 | n.a. | 18 |  | 22 | n.a. | 122 |
| Iron-poor region | 2 | n.a. | 65 |  | 36 | n.a. | 131 |
| Total region | 3 | n.a. | 83 |  | 58 | n.a. | 253 |
|  |  |  |  |  |  |  |  |
| number of records (ninespine) |  |  |  |  |  |  |  |
| Iron-rich region | n.a. | 312 | 23 |  | n.a. | 120 | 324 |
| Iron-poor region | n.a. | 77 | 62 |  | n.a. | 77 | 117 |
| Total region | n.a. | 389 | 85 |  | n.a. | 197 | 441 |


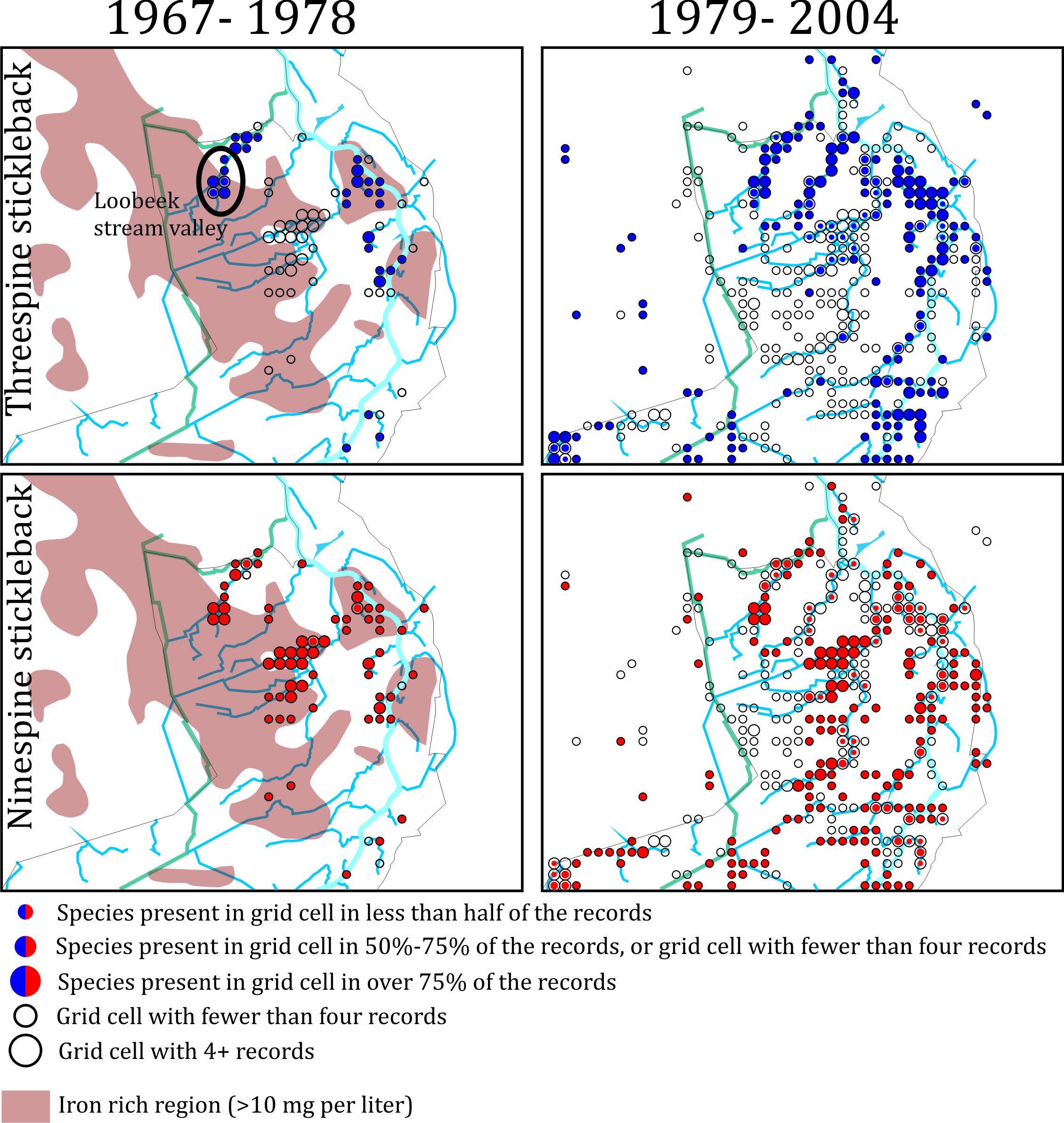


Figure S1.

Distribution of the threespine stickleback (blue, upper) and ninespine stickleback (red, lower) in the Northern Peelregion in two periods (1967-1978; 1979-2003). Parts influenced by iron-rich groundwater are indicated in the left figures. Larger circles indicate more sampling occasions for that grid cell, and the degree of filling corresponds to the number of occasions where a given species of stickleback was observed. Thus ‘empty’ circles indicate sampling did occur but did not yield any sticklebacks, half filled circles indicate sticklebacks were observed but not on all sample occasions, whereas filled circles indicate sticklebacks were observed every time. These ‘empty’ grid cells were typically occupied by common and

widespread fish species such as stone loach (*Barbatula barbatulus* [L 1758]) and in the

second time period also by gudgeon (*Gobio gobio* [L 1758]). The Loobeek stream valley is indicated with an oval (see text below for further explanation).

The figure reveals strong temporal changes in sympatry between threespine and ninespine stickleback in the Northern Peel region, with the ninespine stickleback being much more dominant in iron-rich subregion during 1967-1978, then showing an increasing sympatry with the threespine stickleback in these same regions during 1979-2004. This temporal change in sympatry is most likely related to changes in water quality in the Northern Peel region. Upon completion of land consolidation projects and stream normalisations projects after 1979, the influence of iron-rich seepage water has been reduced due to increased drainage and by feeding these streams with redirected water from the river Meuse (Soesbergen *et al.*, 1990; Verberk *et al.*, 2004a). As a result, harsh conditions were attenuated, reducing the competitive advantage of the ninespine stickleback while allowing the threespine stickleback and other species to expand into these formerly iron-rich areas. For example, also gudgeon (*Gobio gobio*) increased dramatically, from 1 record in the Northern Peel region in 1967-1978 to 296 records in 1979-2004 (Verberk *et al.*, 2004b). Absence of harsh conditions also explains the absence of ninespine stickleback in other parts of the Provence of Limburg (Fig. 1). Ninespine stickleback is notably absent throughout most parts of Southern Limburg, where (i) the substrate is characterised by calcareous marine deposits leading to high calcium levels that reduce the toxicity of heavy metals and (ii) the streams are subject to a higher elevation change resulting in higher flow rates which in turn enhance oxygenation. A similar mechanism explains the apparent exception at the Loobeek stream valley (Fig. 2). Here, a water impermeable deposit lithographic layer called the Asten formation is not present in the subsoil. As a result, strong seepage of deep calcareous groundwater occurs, offsetting any iron toxicity for threespine stickleback in the stream itself and which has long prevented land cultivation (Hellings, 1958).

Figure S2.

Sampling effort and intensity for the Northern Peel region in both time periods. Sampling effort is expressed either as the total number of sampling occasions and from the total number of sampled grid cells. The number of sampling occasions averaged per grid cell is used as a measure of sampling intensity.

Sampling intensity was not directly available in the dataset. However, multiple records for a single species indicate repeated sampling in that grid cell. On this basis, the minimum number of sampling occasions as a measure of sampling intensity was derived for each grid cell for both time periods. For the Northern Peel region, sampling effort was greater in the second period, both when estimated from the total number of sampling occasions and from the total number of sampled grid cells (Figure S2). Consequently, the number of sampling occasions averaged per grid cell, was higher in the first period (1967-1978: 8.5 sampling occasions per grid cell; 1979-2004: 4.7 sampling occasions per grid cell). The distribution of the sampling intensity across grid cells was similar in both time periods (Figure S3). The difference in sampling intensity was used to correct the changes from the first to the second period in the average number of records per grid cell for each species (Fig S4).

Figure S3.

Frequency distribution plots of the sampling intensity in the Northern Peel region in both time periods. Grid cells are categorized according to the minimum number of sampling occasions for that grid cell. Number of grid cells within each category is expressed as a percentage of the total number for that time period. The distribution of the sampling intensity across grid cells was broadly similar in both time periods.

Figure S4.

Average number of records per grid cell for both species in both time periods as a measure of their abundance. Percentual change in average number of records are indicated. These percentual changes are corrected for the 1.8 fold lower sampling intensity in the second time period. Three-spined stickleback avoids the iron rich region (higher number of records per grid cell) in the first period, but expands into this region following intake of (iron-poor) water from the River Meuse. Nine-spined stickleback prefers the iron rich region (higher number of records per grid cell) in both periods, but reductions are largest in the iron rich region following the invasion of three-spined stickleback.

The three-spined stickleback shows an increase in the average number of records, especially in the iron rich region. The pattern is reversed for nine-spined stickleback. These changes in measures of their abundance corroborate the response of both species that is reported in the paper where changes in occupied grid cells and number of records are documented for each species following the inlet of water from the River Meuse (poor in iron).

**Literature**

Hellings, A. (1958). De landbouwwaterhuishouding in de provincie Limburg. Commissie Onderzoek Landbouwwaterhuishouding in Nederland. Delft: TNO.

Soesbergen, M. F., Heinis, F. & Winkel, E. t. (1990). Effecten van de aanvoer van gebiedsvreemd water op aquatische- en terrestrische ecosystemen in Noord-Limburg ten westen van de Maas. Amsterdam: M & W Aquasense.

Verberk, W. C. E. P., Pollux, B. J. A. & Munckhof, P. J. J. v. d. (2004a). Veranderingen in het beekdallandschap van de peelregio Deel I: Een ecologische analyse voor de Driedoornige stekelbaars, de Tiendoornige stekelbaars en het Bermpje. *Natuurhistorisch Maandblad* **93**, 301-310.

Verberk, W. C. E. P., Munckhof, P. J. J. v. d. & Pollux, B. J. A. (2004b). Veranderingen in het beekdallandschap van de peelregio Deel II: Grenzen aan het verspreidingsgebied in Limburg van de driedoornige stekelbaars, de tiendoornige stekelbaars en het bermpje. *Natuurhistorisch Maandblad* **93**, 328-333.
